# Impact of Curcumin (with or without Piperine) on the Pharmacokinetics of Tamoxifen

**DOI:** 10.3390/cancers11030403

**Published:** 2019-03-22

**Authors:** Koen G.A.M. Hussaarts, Daan P. Hurkmans, Esther Oomen-de Hoop, Leonie J. van Harten, Stan Berghuis, Robbert J. van Alphen, Leontine E.A. Spierings, Quirine C. van Rossum-Schornagel, Mijntje B. Vastbinder, Ron H.N. van Schaik, Teun van Gelder, Agnes Jager, Roelof W.F. van Leeuwen, Ron H.J. Mathijssen

**Affiliations:** 1 Department of Medical Oncology, Erasmus MC Cancer Institute, Dr. Molewaterplein 40, 3015 GD Rotterdam, The Netherlands; d.hurkmans@erasmusmc.nl (D.P.H); e.oomen-dehoop@erasmusmc.nl (E.O.-d.H.); l.vanharten@erasmusmc.nl (L.J.v.H.); s.berghuis@erasmusmc.nl (S.B.); mvastbinder@ysl.nl (M.B.V.); a.jager@erasmusmc.nl (A.J.); r.w.f.vanleeuwen@erasmusmc.nl (R.W.F.v.L.); a.mathijssen@erasmusmc.nl (R.H.J.M.); 2 Department of Internal Medicine, Elisabeth Tweesteden Hospital, Hilvarenbeekseweg 60, 5022 GC Tilburg, The Netherlands; r.vanalphen@etz.nl; 3 Department of Internal Medicine, Alrijne Hospital, Simon Smitweg 1, 2353 GA Leiderdorp, The Netherlands; leaspierings@alrijne.nl; 4 Department of Internal Medicine, Franciscus Gasthuis & Vlietland, Kleiweg 500, 3045 PM Rotterdam, The Netherlands; q.vanrossum@franciscus.nl; 5 Department of Clinical Chemistry, Erasmus MC, Dr. Molewaterplein 40, 3015 GD Rotterdam, The Netherlands; r.vanschaik@erasmusmc.nl; 6 Department of Hospital Pharmacy, Erasmus MC, Dr. Molewaterplein 40, 3015 GD Rotterdam, The Netherlands; t.vangelder@erasmusmc.nl

**Keywords:** tamoxifen, curcumin, piperine, pharmacokinetics, drug interactions

## Abstract

Tamoxifen is a prodrug that is primarily metabolized into the pharmacologically active metabolite endoxifen and eventually into inactive metabolites. The herb curcumin may increase endoxifen exposure by affecting phase II metabolism. We compared endoxifen and tamoxifen exposure in breast cancer patients with or without curcumin, and with addition of the bio-enhancer piperine. Tamoxifen (20–30mg per day (q.d.)) was either given alone, or combined with curcumin (1200 mg three times daily (t.i.d.)) +/− piperine (10 mg t.i.d.). The primary endpoint of this study was the difference in geometric means for the area under the curve (AUC) of endoxifen. Genotyping was performed to determine CYP2D6 and CYP3A4 phenotypes. The endoxifen AUC_0–24h_ decreased with 7.7% (95%CI: −15.4 to 0.7%; *p* = 0.07) with curcumin and 12.4% (95%CI: −21.9 to −1.9%; *p* = 0.02) with curcumin and piperine, compared to tamoxifen alone. Tamoxifen AUC_0–24h_ showed similar results. For patients with an extensive CYP2D6 metabolism phenotype (EM), effects were more pronounced than for intermediate CYP2D6 metabolizers (IMs). In conclusion, the exposure to tamoxifen and endoxifen was significantly decreased by concomitant use of curcumin (+/− piperine). Therefore, co-treatment with curcumin could lower endoxifen concentrations below the threshold for efficacy (potentially 20–40% of the patients), especially in EM patients.

## 1. Introduction

Breast cancer is one of the most commonly diagnosed malignancies worldwide and one of the leading causes of cancer-related deaths in women [1]. For decades, patients with estrogen receptor-positive breast cancer have been extensively treated with endocrine therapy such as tamoxifen. Tamoxifen acts as a selective estrogen receptor modulator in breast cancer tissue, thereby reducing the risk of disease recurrence and breast cancer-specific mortality [2].

Currently, there is a trend towards the use of natural herbs and dietary products among cancer patients. Nearly 20–30% of all cancer patients, especially breast cancer patients, use herbal medicine besides their conventional therapy [3]. Curcumin, also called “turmeric”, a spice recovered from the roots of the *Curcuma longa* plant, is becoming increasingly popular among cancer patients because of its supposed anti-cancer effects [4]. Curcumin is characterized by a poor bioavailability because of its poor absorption and rapid metabolism [5]. Therefore, curcumin is often used in combination with piperine (a component of black pepper). Piperine increases curcumin bioavailability 20-fold by increasing curcumin absorption and inhibition of curcumin glucuronidation [6].

**Figure 1:**
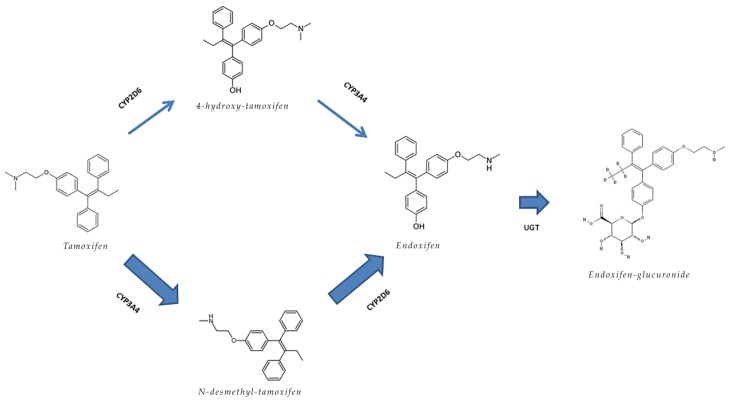
The major primary metabolite *N*-desmethyl-tamoxifen and the minor primary metabolite 4-hydroxytamoxifen are formed by *N*-demethylation and 4-hydroxylation of tamoxifen, through CYP3A4 and CYP2D6 metabolism, respectively. Further CYP-mediated metabolism of these metabolites results in the formation of 4-hydroxy-*N*-desmetyltamoxifen (endoxifen). Endoxifen is ultimately metabolized through phase II metabolism into among others endoxifen–glucoronide through UDP-glucuronyltransferases (UGTs) and also through sulfotransferase (SULT) enzymes.

Tamoxifen shows a complex and multi-pathway metabolism, which mainly occurs in the liver. Tamoxifen is metabolized into several (active) metabolites, through several phase I and phase II metabolizing enzymes, mainly by the cytochrome P450 (CYP) enzymes CYP2D6 and CYP3A4 (Figure 1) [7]. Based on its relatively high plasma concentrations and potency, endoxifen is believed to be one of the most important metabolites in the efficacy of tamoxifen therapy [7,8]. Moreover, endoxifen is excreted (mainly in the feces) after phase II metabolism through UDP-glucuronyltransferases (UGTs) and sulfotransferase (SULT) [8]. 

A study in rats demonstrated an increase in tamoxifen plasma concentration of 33–64%, suggesting an inhibitory effect of curcumin on tamoxifen metabolism [9]. Several studies (both in vitro and in vivo) demonstrated that curcumin has an inhibitory effect on several CYP enzymes, such as CYP3A4 and CYP2D6 [10]. Another important effect of curcumin is its inhibition of phase II drug metabolism by inhibition of UGT. Furthermore, curcumin could potentially inhibit or induce several drug-efflux transporters (e.g., P-glycoprotein (P-gp), which may be inhibited or induced) [10,11,12]. 

In this study, it was hypothesized that endoxifen plasma concentrations may increase when tamoxifen is administered with curcumin, mainly through UGT enzyme inhibition. In addition, concomitant administration with the bio-enhancer piperine may potentiate effects on tamoxifen and endoxifen plasma pharmacokinetics. In a pharmacokinetic cross-over study we therefore explored the impact of curcumin—with and without piperine—on tamoxifen and endoxifen pharmacokinetics.

## 2. Results

### 2.1. Patient Characteristics

Seventeen patients were included in the study. One patient was excluded because of voluntary withdrawal, resulting in 16 evaluable patients. Patient characteristics can be found in Table 1. DNA analysis showed no variants for CYP3A4*22. As this polymorphism is considered most relevant for CYP3A4, a predicted normal CYP3A4 phenotype for all study patients based on genotype was assumed [13,14]. Based on CYP2D6 genotyping, seven patients (44%) showed an extensive CYP2D6 metabolism phenotype (EM), while seven other patients (44%) exhibited intermediate CYP2D6 metabolism (IM). The other two patients (12%) demonstrated ultra-rapid metabolism (UM) and poor CYP2D6 metabolism (PM), respectively.

### 2.2. Pharmacokinetics

In patients treated with tamoxifen and curcumin, the geometric mean AUC_0–24h_ and C_trough_ of tamoxifen decreased 8.0% (95%CI: −14.1% to −1.4%, *p* = 0.02) and 7.1% (95%CI: −17.1% to 4.0%, *p* = 0.25), respectively, compared to tamoxifen monotherapy (Table 2). Furthermore, AUC_0–24h_ and C_trough_ of endoxifen decreased 7.7% (95%CI: −15.4% to 0.7%, *p* = 0.07), and 5.6% (95%CI: −15.6% to 5.5%, *p* =0.43), respectively, with concomitant curcumin treatment.

When tamoxifen was administered with curcumin and piperine, the effects were more pronounced; tamoxifen AUC_0–24h_ and C_trough_ decreased 12.8% (95%CI: −19.2% to −5.9%, *p* < 0.01) and 12.2% (95%CI: −21.5% to −1.8%, *p* = 0.02), respectively, compared to tamoxifen monotherapy. The endoxifen AUC_0–24h_ decreased 12.4% (95%CI: −21.9% to −1.9%, *p* = 0.02), while the C_trough_ decreased 12.4% (95%CI: −20.9% to −3.0%, *p* =0.01). Further pharmacokinetic results are shown in Table 2. Pharmacokinetic parameters for 4-hydroxy-tamoxifen and *N*-desmethyl tamoxifen showed a decrease in almost every pharmacokinetic parameter when administered with curcumin—with and without piperine—although only AUC_0–24h_ of *N*-desmethyl tamoxifen and 4-hydroxytamoxifen with curcumin and AUC_0–24h_ of *N*-desmethyl tamoxifen with curcumin and piperine demonstrated a significant difference.

**Figure 2:**
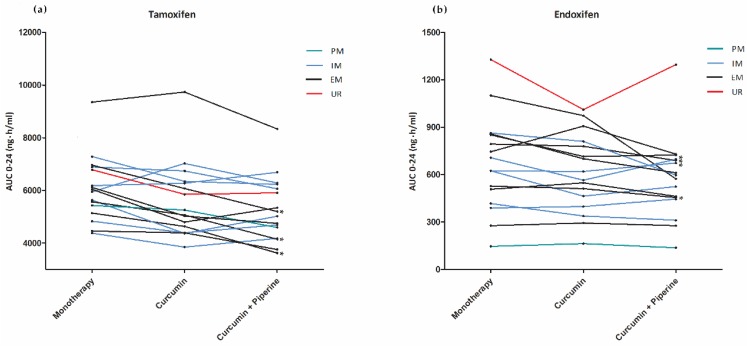
Endoxifen and tamoxifen AUC_0–24h_ per individual patient per treatment phase: (**a**) Tamoxifen AUC_0–24h_ per individual patients per treatment phase. (**b**) endoxifen AUC_0–24h_ per individual patients per treatment phase. Patients with an intermediate CYP2D6 metabolism (IM) were colored blue. Patients with an extensive CYP2D6 metabolism (EM) were colored black. Poor CYP2D6 metabolizers (PM) and ultra-rapid CYP2D6 metabolizers (UR) were colored green and red, respectively; *: decrease in AUC_0–24h_ >25%; a total of four patients showed a >25% decrease in endoxifen AUC_0–24h_ and three patients in tamoxifen AUC_0–24h_ when tamoxifen was administered with curcumin and piperine, compared to tamoxifen monotherapy.

When analyzing the CYP2D6-predicted phenotypes separately, both endoxifen and tamoxifen showed a more pronounced decrease for both AUC_0–24h_ and C_trough_ (especially during treatment with curcumin and piperine) in patients with an extensive metabolism, compared to those with an intermediate metabolism (Table 3). In patients with an IM treated with curcumin and piperine, the AUC_0–24h_ decreased 5.3% (95%CI: −13.1% to +3.1%, *p* = 0.16) and 10.3% (95%CI: −23.5% to 5.3%, *p* = 0.14) for tamoxifen and endoxifen, respectively. In patients with an EM treated with curcumin and piperine, the tamoxifen and endoxifen AUC_0–24h_ decreased 22.0% (95%CI: −29.0% to −14.2%, *p* < 0.01) and 18.4% (95%CI: −36.1% to 4.3%, *p* = 0.09), respectively. C_trough_ showed similar results (Table 3). Although, the interaction term was only significant for tamoxifen AUC_0–24h_ and C_trough_ with curcumin and piperine. There was no period effect, which implicated no decline in tamoxifen nor endoxifen plasma concentrations based on altered tamoxifen metabolism over time. Individual tamoxifen and endoxifen AUC_0–24h_ can be found in Figure 2 and in Supplementary Figure S1.

### 2.3. Toxicities

There were no unexpected serious adverse events (SAE) during combined treatment with curcumin or curcumin plus piperine related to the study procedures. There was one serious adverse event, which was assumed to be not related to any of the study drugs (collapse with unknown origin). Toxicity profiles were similar between the different treatment phases, although more hot flashes and fatigue were observed in patients treated with curcumin +/− piperine compared to tamoxifen monotherapy (Table 4). Interestingly, three patients suffered from grade 2–3 diarrhea during treatment with curcumin and piperine, whereas none of the patients experienced diarrhea when treated with tamoxifen monotherapy.

## 3. Discussion

In this study, a modest but significant decrease was found in both tamoxifen and endoxifen plasma concentrations during concomitant administration of tamoxifen and curcumin, compared to tamoxifen monotherapy. This effect was even more pronounced when tamoxifen was administered with curcumin and piperine. Furthermore, patients with an extensive CYP2D6 phenotype seem to be at greater risk of experiencing this herb–drug interaction, compared to CYP2D6 intermediate metabolizers.

Since tamoxifen metabolism and excretion is complex and involves multiple enzymes and transporter proteins, the likelihood of a drug–drug interaction (DDI) with modulators and inhibitors of enzymes and drug-transporters (e.g., CYP3A4 and P-glycoprotein) involved in tamoxifen metabolism is high [7,8]. Based on preclinical data, curcumin is a compound that could potentially lead to such a DDI [10]. When designing our study, we based our hypothesis on preclinical data, as no studies in cancer patients studying the effects of curcumin on the pharmacokinetics of anti-cancer drugs were available in the literature. Cho et al. demonstrated an increase in tamoxifen exposure and a decrease in 4-hydroxy-tamoxifen/tamoxifen AUC ratio, suggesting a decrease in CYP-mediated metabolism or P-glycoprotein-mediated efflux of tamoxifen [9]. On the other hand, in vitro results indicate an inhibitory effect of curcumin on phase II metabolism, in which enzymes such as UGT are involved [10,15]. UGTs are also involved in the metabolism of tamoxifen (Figure 1), in theory resulting in increased endoxifen plasma concentrations [15]. In contrast to the study in rats by Cho et al., we found both a decrease in tamoxifen and endoxifen plasma concentrations. We do not have a conclusive explanation for this observation, as several mechanisms might be involved. One of the limitations of this study is that we could not further explore the mechanisms of this interaction. However, as endoxifen plasma concentrations did not increase during concomitant treatment with curcumin and piperine, an inhibitory effect on phase II metabolism is unlikely, as these results rather indicate phase II induction. A more likely explanation is inhibition of CYP2D6, which is underlined by the larger effect in EM patients. Although, this cannot be the only explanation, as tamoxifen concentrations also decreased due to curcumin co-treatment, where an increase was expected if there was solely an inhibitory effect on CYP2D6. Another explanation may be found in a potential interaction with P-glycoprotein. This transporter, which is responsible for the efflux of tamoxifen out of the epithelial cells into the gut and bile, was studied before in vitro and led to contrasting results (but especially inhibition) [10,12,16]. However, in case curcumin acts as a P-glycoprotein inducer, tamoxifen and metabolite plasma concentrations would all decrease, which is in line with our findings, resulting from a diminished absorption of tamoxifen into the blood stream. Yet, the evidence supporting curcumin as a P-glycoprotein inducer is limited.

One of the main problems of clinical research with curcumin is to standardize the formulation of curcumin. There are many curcumin formulations available. Potentially these formulations may differ in bio-availability, which makes it difficult to determine the individual impact of these formulations and to give general advisories [17]. Moreover, many of these formulations exist of multiple non-standardized ingredients. In this study, standardized formulations of both curcumin and piperine, of 1200 mg and 10 mg capsules, respectively, were used from a single production batch.

Moreover, curcumin has low bio-availability, and the suggested inter-patient variability is high [4,18]. We only measured tamoxifen pharmacokinetics and did not determine plasma levels of curcumin, which gives a possible limitation of this study since the magnitude of a possible interaction may differ between patients depending on curcumin plasma levels [18]. However, a curcumin dose of 3.6 g q.d. is considered a significant plasma concentration and is therefore most likely to achieve a significant drug interaction [18].

Furthermore, the interaction term for CYP2D6 metabolism only reached significance for tamoxifen AUC_0–24h_ and C_trough_ when co-administered with curcumin and piperine. Since the design of this study was not sufficient to detect a significant difference in other pharmacokinetic endoxifen and tamoxifen comparisons, this result must be confirmed in future clinical trials.

Besides curcumin, piperine might also influence pharmacokinetics of tamoxifen and endoxifen by itself [19]. Piperine affects the structure of the intestinal lumen and wall, resulting in a higher passive drug influx. Concomitant piperine use might alter gastric emptying time in a dose- and time dependent manner, and in addition, piperine is known to be a P-glycoprotein inhibitor in vivo [19,20]. Moreover, piperine might enhance plasma concentrations of several drugs because of the inhibition of CYP enzymes (e.g., CYP3A4, CYP2D6) [19]. Therefore, the effect of piperine on tamoxifen and endoxifen pharmacokinetics may not be underestimated, and the results in this study could not be solely attributed to curcumin.

The relatively small effects on tamoxifen and endoxifen pharmacokinetics and the high inter-patient variability of endoxifen (CV = 50–60%) in this study may seem of limited clinical relevance. However, individual patients may be deprived from an optimal therapy, since tamoxifen and endoxifen concentrations may drop below the threshold for efficacy (~16 nM for endoxifen) resulting from co-treatment with curcumin [7,21]. This threshold is associated with a survival benefit, and 20–30% of all treated patients had an endoxifen plasma concentration below this threshold, and an additional 20% had endoxifen plasma concentrations just above this threshold [21,22,23]. This scenario is particularly the case in patients with an extensive metabolism CYP2D6 phenotype, as effects of curcumin with and without piperine were most pronounced in this group of patients.

## 4. Materials and Methods 

This two-arm, three-period, randomized, cross-over study was performed between January 2017 and May 2018 at the Erasmus University Medical Center. The study was approved (approval date: 19 January 2017, ethics committee code: METC-16-679) by the local ethics committee of the Erasmus University Medical Center and competent authority in accordance to the declaration of Helsinki, and it was registered at the European Clinical Trials Database (EudraCT 2016-004008-71) and the Dutch trial registry (www.trialregister.nl; number NTR6149).

### 4.1. Patients

We included patients who had a histological or cytological confirmed diagnosis of breast cancer with an indication for tamoxifen treatment who were at least 18 years of age. In addition, an Eastern Cooperative Oncology Group (ECOG) performance status of 0 or 1, and adequate hematological, renal, and liver function (defined as a CTCAE grade of ≤1) were required. Patients were able and willing to abstain from curry, grapefruit (juice), (herbal) dietary supplements, and herbals or over-the-counter medication (except for paracetamol and ibuprofen) for the duration of the study. Patients were excluded if they had known impaired drug absorption (e.g., gastrectomy), serious illness, or medically unstable conditions requiring treatment (e.g., infection, heart failure) or if they used strong CYP3A4, CYP2D6, UGT, or P-glycoprotein inhibitors or inducers. All included patients gave written informed consent.

### 4.2. Study Procedures

Patients received tamoxifen at the same dose for at least 28 days before entering the study to ensure steady-state pharmacokinetics. No dose alterations were allowed after inclusion in the study. Patients were digitally randomly assigned into two sequence groups, using block randomization, to rule out sequence effects. Tamoxifen was administered at a constant dose (20–30 mg q.d.) during three consecutive cycles. During cycle one, patients received tamoxifen monotherapy; in cycle two, patients swallowed tamoxifen concomitant with curcumin (three times daily 1,200 mg), and in cycle three, tamoxifen was taken concomitantly with curcumin and piperine (three times daily 1,200 mg and three times daily 10 mg, respectively) in the first sequence group or vice versa in the other group. Curcumin and piperine were taken at predefined time points (10 AM, 4 PM, and 10 PM). Patient compliance was assessed through a patient diary until the end of study after three consecutive cycles. Furthermore, CYP2D6 and CYP3A4*22 mutational analyses were performed.

### 4.3. Pharmacokinetic Sampling 

Patients were admitted to the hospital on days 28, 56, and 84 of the study for pharmacokinetic blood sampling. Blood samples for determination of tamoxifen, 4-hydroxytamoxifen, n-desmethyl-tamoxifen, and endoxifen pharmacokinetics were obtained at predefined time points (*t* = 0 (before tamoxifen intake); and 0.5 h; 1 h; 1.5 h; 2 h, 2.5 h; 3 h; 3.5 h; 4 h; 6 h; 8 h; 12 h; and 24 h after tamoxifen intake). Blood samples were processed into plasma within 30 minutes by vortex mixing and centrifugation for 10 min at 2500–3000× *g* at 4 °C. Plasma concentrations were measured using a validated liquid chromatography with a tandem mass spectrometry method (UP-LCMS/MS) [24]. Predefined pharmacokinetic parameters were tamoxifen and endoxifen exposure (expressed as dose-corrected area under the curve from the pre-infusion time point until 24h (AUC_0–24h_)), maximum concentration (C_max_), time until maximum concentration (T_max_), and lowest plasma concentration (C_trough_). Pharmacokinetic outcomes were calculated using Phoenix WinNonlin version 8.1 (Pharsight, a Certara Company, Princeton, NJ, USA).

### 4.4. Toxicity

Toxicity rates during tamoxifen monotherapy and tamoxifen concomitantly with curcumin +/− piperine were determined during patient follow-up until the end of the study using Common Terminology Criteria for Adverse Events (CTCAE version 4.0, National Cancer Institute, Bethesda, MD, USA) and by evaluating the patient diaries.

### 4.5. Statistical Analysis

A difference in systemic exposure (AUC_0–24h_) to endoxifen of 25% between treatment cycles was considered to be clinically relevant. It was assumed that the within patient standard deviation in endoxifen pharmacokinetics was 20% [25]. Given a power of 80%, this resulted in a sample size of 16 evaluable patients [26].

Analyses of AUC_0–24h_ , C_trough_, and C_max_ were performed on log-transformed observations since these were assumed to follow a log-normal distribution [27]. Estimates for the mean differences in (log) AUC_0–24h_, C_trough_, and C_max_ were obtained for the two comparisons (i.e., curcumin versus tamoxifen monotherapy, and curcumin plus piperine versus tamoxifen monotherapy) separately using a linear mixed effect model with treatment, sequence, and period as fixed effects and subject within sequence as a random effect [28]. Variance components were estimated based on restricted maximum likelihood (REML) methods, and the Kenward–Roger method of computing the denominator degrees of freedom was used [28]. Since two primary comparisons were made, a Bonferroni correction was applied to correct for multiple testing (two-sided alpha of 5%/2 = 2.5%). T_max_ was analyzed by means of the Wilcoxon signed-rank test and described with medians and interquartile ranges.

For a comparison between extensive and intermediate CYP2D6 phenotypes, an interaction term between metabolism and treatment was added to the linear mixed effects models. Only if the interaction term turned out to be significant, subsequent subgroup analyses were performed.

Toxicity was described as the incidence of toxicity per phase. This was corrected for baseline toxicity and was only taken into account in case of an increase in CTCAE grade per cycle. Since the design of this study was not appropriate to detect a significant difference in toxicity, these results had a descriptive character.

## 5. Conclusions

This was the first study that investigated the influence of curcumin (with and without piperine) on tamoxifen pharmacokinetics. The use of curcumin (with and without piperine) significantly decreased tamoxifen and endoxifen pharmacokinetics, especially in EM patients. Patients may be deprived from optimal tamoxifen treatment, and endoxifen plasma levels may even drop below the threshold for treatment efficacy. Therefore, patients should be advised to stop curcumin use during tamoxifen treatment, or treatment efficacy of tamoxifen should be adequately monitored. 

## Figures and Tables

**Table 1:** Patient characteristics.

Characteristic	N (%)
Patients	16 (100)
Randomization sequence	
- ABC	9 (56)
- CBA	7 (44)
Adjuvant tamoxifen treatment	16 (100)
Age (Median, IQR)	45 (42–58)
Sex	
- Female	15 (94)
- Male	1 (6)
Race	
- Caucasian	15 (94)
- Arabic	1 (6)
Height (Median, IQR)	171 (167–176)
Weight (Median, IQR)	73 (65–91)
BMI (Median, IQR)	25 (23–29)
WHO Performance Status	
- 0	13 (81)
- 1	3 (19)
Previous chemotherapy	
- Yes	12 (75)
○ TAC	2 (13)
○ AC - paclitaxel	4 (25)
○ FEC - docetaxel	6 (37)
- No	4 (25)
Previous RTx	
- Yes	10 (63)
- No	6 (37)
Tamoxifen dose	
- 20 mg	15 (94)
- 30 mg	1 (6)
Genotype	
- CYP3A4*22	
○ EM	16 (100)
- *CYP2D6*	
○ EM	7 (44)
○ IM	7 (44)
○ PM	1 (6)
○ UM	1 (6)

Abbreviations: IQR = interquartile range; TAC = Docetaxel, doxorubicin, and cyclofosfamide; AC = doxorubicin and cyclofosfamide; FEC = 5FU, epirubicine, and cyclofosfamide; RTx = radiotherapy; EM = extensive metabolism phenotype; IM = intermediate metabolism phenotype; PM = poor metabolism phenotype; UM = ultra-rapid metabolism phenotype.

**Table 2:** Tamoxifen pharmacokinetics.

PK Parameters	Tamoxifen Monotherapy (A)	Tamoxifen + Curcumin (B)	Tamoxifen + Curcumin + Piperine (C)	Relative Difference A vs B (95%CI)	*p*-Value	Relative Difference (A vs C) (95%CI)	*p*-Value
**Tamoxifen**							
AUC_0–24h_	5951 (20)	5460 (24)	5171 (23)	−8.0% (−14.1 to −1.4)	0.02	−12.8% (−19.2 to −5.9)	<0.01
C_trough_	213 (27)	198 (28)	187 (24)	−7.1% (−17.1 to +4.0)	0.25	−12.2% (−21.5 to −1.8)	0.02
C_max_	356 (16)	324 (21)	313 (22)	−8.4% (−16.4 to +0.5)	0.07	−11.1% (−18.1 to −3.6)	<0.01
T_max_	2.4 (1.9 to 3.1)	2.4 (1.9 to 3.0)	2.7 (1.9 to 3.8)		0.74		0.34
**Endoxifen**							
AUC_0–24h_	597 (59)	556 (52)	518 (54)	−7.7 % (−15.4 to +0.7)	0.07	−12.4% (−21.9 to −1.9)	0.02
C_trough_	25 (60)	23 (53)	21 (55)	−5.6 % (−15.6 to +5.5)	0.43	−12.4% (−20.9 to −3.0)	0.01
C_max_	31 (56)	28 (50)	27 (51)	−7.1% (−16.3 to +3.2)	0.20	−9.8% (−20.1 to +1.8)	0.10
T_max_	2.0 (1.3 to 3.0)	1.7 (1.2 to 2.6)	1.8 (1.1 to 3.1)		0.88		0.62
**4-hydroxy-tamoxifen**							
AUC_0–24h_	113 (31)	106 (24)	103 (28)	−6.3 % (−11.6 to −0.73)	0.03	−8.2% (−17.0 to +1.6)	0.11
C_trough_	4.4 (34)	4.2 (26)	4.1 (28)	−4.3 % (−12.3 to +4.4)	0.45	−7.3 (−17.6 to +4.3)	0.26
C_max_	6.0 (32)	5.4 (26)	5.4 (31)	−10.0% (−16.8 to −2.6)	<0.01	−8.8% (−20.0 to +4.0)	0.20
T_max_	2.7 (1.9 to 3.9)	2.4 (1.8 to 3.3)	2.8 (2.0 to 3.8)		0.42		0.37
***N*** **-desmethyl-tamoxifen**							
AUC_0–24h_	11596 (21)	10766 (24)	10084 (31)	−7.0% (−13.1 to −0.6)	0.03	−12.4% (−22.3 to −1.3)	0.03
C_trough_	463 (28)	430 (29)	411 (32)	−7.2% (−15.0 to +1.2)	0.10	−10.9% (−21.6 to +1.3)	0.08
C_max_	602 (21)	556 (24)	540 (32)	−7.2% (−14.9 to +1.1)	0.09	−9.7% (−20.2 to +2.3)	0.12
T_max_	2.6 (1.8 to 3.7)	1.7 (1.2 to 2.4)	2.1 (1.4 to 3.2)		0.24		0.88

Abbreviations: PK = pharmacokinetics; CI = Confidence Interval; AUC_0–24h_= area under the curve, timepoint 0 h to 24 h (expressed as geomean nM·h/mL (CV%)); C_trough_= minimum concentration (expressed as geomean nM/mL (CV%)); C_max_= maximum concentration (expressed as geomean nM/mL (CV%)); T_max_= time until maximum concentration (expressed as median h (IQR)); CV% = coefficient of variation; IQR = interquartile range.

**Table 3:** Tamoxifen pharmacokinetics, based on CYP2D6 phenotype.

PK Parameters	Tamoxifen Monotherapy (A)	Tamoxifen + Curcumin (B)	Tamoxifen + Curcumin + Piperine (C)	Relative Difference A vs B (95%CI)	*p*-Value	Relative Difference A vs C (95%CI)	*p*-Value
**Intermediate Metabolizers (IM)**							
Tamoxifen AUC_0–24h_	5795 (4895–6859)	5427 (4313–6830)	5518 (4679–6508)	−7.2% (−18.2 to +5.4)	0.19	−5.3% (−13.1 to +3.1)	0.16 *
Tamoxifen C_trough_	200 (160–251)	191 (146–249)	199 (167–237)	−5.9% (−20.9to +11.9)	0.41	−1.3% (−15.3 to 15.1)	0.84 *
Endoxifen AUC_0–24h_	523 (362–755)	472 (339–656)	477 (340–669)	−9.4% (−21.7 to +4.8)	0.14	−10.3% (−23.5 to 5.3)	0.14
Endoxifen C_trough_	21 (14–32)	19 (13–27)	19 (14–27)	−10.7% (−28.2 to 11.2)	0.24	−8.3% (−27.2 to 15.4)	0.38
**Extensive Metabolizers (EM)**							
Tamoxifen AUC_0–24h_	6077 (4882–7565)	5471 (4247–7047)	4836 (3720–6288)	−10.3% (−19.7 to +0.3)	0.06	−22.0% (−29.0 to −4.2)	<0.01 *
Tamoxifen C_trough_	218 (163–291)	199 (148–268)	170 (132–218)	−9.6% (−26.4 to +11.2)	0.27	−24.6% (−33.9 to −14.1)	<0.01 *
Endoxifen AUC_0–24h_	745 (576–963)	716 (574–893)	596 (495–717)	−5.7% (−19.6 to +10.7)	0.39	−18.4% (−36.1 to +4.3)	0.09
Endoxifen C_trough_	30 (23–39)	31 (25–38)	25 (20–30)	−0.3% (−12.8 to +13.9)	0.96	−17.2% (−26.1 to −7.3)	<0.01 *

Abbreviations: PK = pharmacokinetics; CI = Confidence Interval; AUC_0–24h_= Area under the curve, timepoint 0 h to 24 h (expressed as geomean nM·h/mL (95%CI)); C_trough_= minimum concentration (expressed as geomean nM/mL (95%CI)); * Interaction term reached statistical significance (*p* < 0.05).

**Table 4:** Adverse Events.

Adverse Event	Tamoxifen Monotherapy *N* (%) (A)	Tamoxifen with Curcumin *N* (%) (B)	Tamoxifen with Curcumin and Piperine *N* (%) (C)
Nausea	2(13)	1(6)	1(6)
Diarrhea	0	1(6)	3(19)
Constipation	2(13)	4(25)	1(6)
Fatigue	2(13)	3(19)	3(19)
Hot flashes	3(19)	5(31)	4(25)
Reflux	1(6)	1(6)	0
Dyspnea	0	1(6)	0
Anorexia	1(6)	0	1(6)
Pain	4(25)	0	2(13)
Rash	1(6)	0	1(6)
Hypophosphatemia	0	0	1(6)
Hyperlipidemia	1(6)	1(6)	1(6)

Legend: Number of patients with all Common Terminology Criteria for Adverse Events (CTCAE)-grade adverse events when treated with tamoxifen with or without curcumin and piperine expressed as number of patients (% of total number of patients).

## Supplementary Materials

The following are available online at https://www.mdpi.com/2072-6694/11/3/403/s1, Figure S1: Time-concentration (AUC) curves for both tamoxifen (A) and endoxifen (B) for every individual patient (coded as patient 001–016).
