# Supplementary material for: Impact of Curcumin (with or without Piperine) on the Pharmacokinetics of Tamoxifen
Source: Cancers (Basel). 2019 Mar 22;11(3):403. doi: 10.3390/cancers11030403 (PMC6468355; doi:10.3390/cancers11030403)
Supplement: Supplementary file 1 [file cancers-11-00403-s001.pdf]

Supplementary Material: Impact of Curcumin (with or without Piperine) on the Pharmacokinetics of Tamoxifen

Koen G.A.M. Hussaarts, Daan P. Hurkmans, Esther Oomen-de Hoop, Leonie J. van Harten, Stan Berghuis, Robbert J. van Alphen, Leontine E.A. Spierings, Quirine C. Van Rossum-Schornagel, Mijntje B. Vastbinder, Ron H.N. van Schaik, Teun van Gelder, Agnes Jager, Roelof W.F. van Leeuwen and Ron H.J. Mathijssen

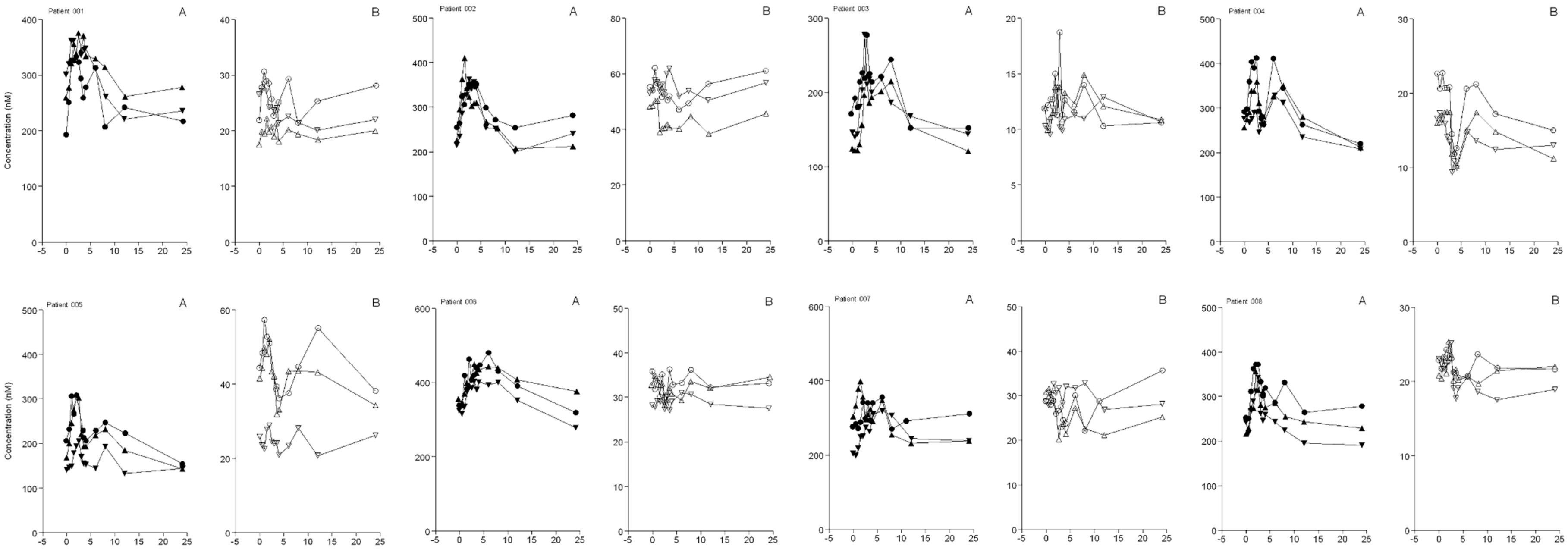

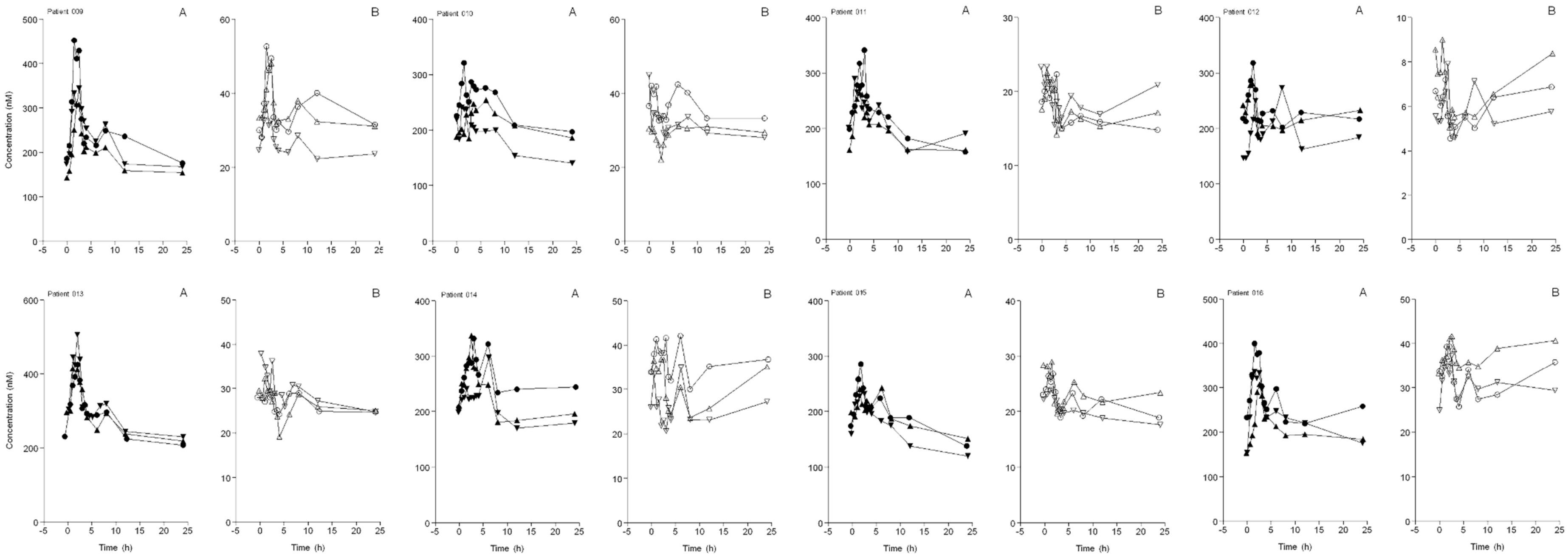

Figure 1. Time-concentration (AUC) curves for both tamoxifen (A) and endoxifen (B) for every individual patient (coded as patient 001–016). Curves were displayed as ● = Phase A (tamoxifen monotherapy), ▲ = Phase B (tamoxifen concomitantly with curcumin) or ▼= Phase C (tamoxifen concomitantly with curcumin and piperine). Individual patient characteristics for every individual patient were: Patient 001: randomization arm: CBA; age: 42 years; sex: female; race: Caucasian; height: 166 cm; weight: 64 kg; BMI: 23.2 kg/m^2; WHO performance status: 0 ; previous chemotherapy: yes (TAC); previous radiotherapy: yes; tamoxifen dose: 20 mg; CYP2D6 genotype: IM Patient 002: randomization arm: CBA; age: 28 years; sex: female; race: Asian; height: 166 cm; weight: 64.4 kg; BMI: 23.4 kg/m^2; WHO performance status: 1 ; previous chemotherapy: no; previous radiotherapy: no; tamoxifen dose: 20 mg; CYP2D6 genotype: UR; Patient 003: randomization arm: ABC; age: 48 years; sex: female; race: Caucasian; height: 178 cm; weight: 79.8 mg; BMI: 25.2 kg/m^2; WHO performance status: 0; previous chemotherapy: yes (AC-paclitaxel); previous radiotherapy: no; tamoxifen dose: 20 milligrams; CYP2D6 genotype: IM; Patient 004: randomization arm: ABC; age: 44 years; sex: female; race: Caucasian; height: 170 cm; weight: 95 mg; BMI: 32.9 kg/m^2; WHO performance status: 0 ; previous chemotherapy: yes (FEC-docetaxel); previous radiotherapy: yes tamoxifen dose: 30 milligrams; CYP2D6 genotype: IM; Patient 005: randomization arm: ABC; age: 67 years; sex: female; race: Caucasian; height: 173 cm; weight: 75.7 mg; BMI: 25.3 kg/m^2; WHO performance status: 0 ; previous chemotherapy: yes (FEC-docetaxel); previous radiotherapy: yes tamoxifen dose: 20 milligrams; CYP2D6 genotype: EM; Patient 006: randomization arm: ABC; age: 56 years; sex: female; race: Caucasian; height: 180 cm; weight: 103 mg; BMI: 31.8 kg/m^2; WHO performance status: 0 ; previous chemotherapy: yes (FEC-docetaxel); previous radiotherapy: yes tamoxifen dose: 20 milligrams; CYP2D6 genotype: EM; Patient 007: randomization arm: CBA; age: 39 years; sex: female; race: Caucasian; height: 169 cm; weight: 66.5 mg;

BMI: 23.3 kg/m^2; WHO performance status: 0 ; previous chemotherapy: yes (FEC-docetaxel); previous radiotherapy: yes tamoxifen dose: 20 milligrams; CYP2D6 genotype: IM; Patient 008: randomization arm: CBA; age: 36 years; sex: female; race: Caucasian; height: 167 cm; weight: 98 mg; BMI: 35.1 kg/m^2; WHO performance status: 0 ; previous chemotherapy: no; previous radiotherapy: no tamoxifen dose: 20 milligrams; CYP2D6 genotype: EM; Patient 009: randomization arm: ABC; age: 56 years; sex: female; race: Caucasian; height: 167 cm; weight: 62.4 mg; BMI: 22.4 kg/m^2; WHO performance status: 1 ; previous chemotherapy: no; previous radiotherapy: yes tamoxifen dose: 20 milligrams; CYP2D6 genotype: IM; Patient 010: randomization arm: CBA; age: 58 years; sex: female; race: Caucasian; height: 177 cm; weight: 82 mg; BMI: 26.1 kg/m^2; WHO performance status: 0 ; previous chemotherapy: yes (AC-paclitaxel); previous radiotherapy: yes tamoxifen dose: 20 milligrams; CYP2D6 genotype: EM; Patient 011: randomization arm: CBA; age: 45 years; sex: female; race: Caucasian; height: 172 cm; weight: 79 mg; BMI: 26.7 kg/m^2; WHO performance status: 0 ; previous chemotherapy: yes (FEC-docetaxel); previous radiotherapy: yes tamoxifen dose: 20 milligrams; CYP2D6 genotype: IM; Patient 012: randomization arm: ABC; age: 42 years; sex: female; race: Caucasian; height: 178 cm; weight: 94 mg; BMI: 29.7 kg/m^2; WHO performance status: 1 ; previous chemotherapy: yes (AC-paclitaxel); previous radiotherapy: yes tamoxifen dose: 20 milligrams; CYP2D6 genotype: PM; Patient 013: randomization arm: CBA; age: 43 years; sex: female; race: Caucasian; height: 172 cm; weight: 67.1 mg; BMI: 22.7 kg/m^2; WHO performance status: 0 ; previous chemotherapy: yes (AC-paclitaxel); previous radiotherapy: no tamoxifen dose: 20 milligrams; CYP2D6 genotype: IM; Patient 014: randomization arm: ABC; age: 74 years; sex: male; race: Caucasian; height: 166 cm; weight: 64.1 mg; BMI: 23.3 kg/m^2; WHO performance status: 0; previous chemotherapy: yes (FEC-docetaxel); previous radiotherapy: yes; tamoxifen dose: 20 milligrams; CYP2D6 genotype: EM; Patient 015: randomization arm: ABC; age: 43 years; sex: female; race: Caucasian; height: 172 cm; weight: 68.5 mg; BMI: 23.1 kg/m^2; WHO performance status: 0; previous chemotherapy: yes (TAC); previous radiotherapy: no; tamoxifen dose: 20 milligrams; CYP2D6 genotype: EM; Patient 016: randomization arm: ABC; age: 58 years; sex: female; race: Caucasian; height: 167 cm; weight: 70 mg; BMI: 25.1 kg/m^2; WHO performance status: 0; previous chemotherapy: no; previous radiotherapy: no; tamoxifen dose: 20 milligrams; CYP2D6 genotype: EM.
